# Supplementary material for: The histone demethylase Kdm6b regulates subtype diversification of mouse spinal motor neurons during development
Source: Nat Commun. 2022 Feb 17;13:958. doi: 10.1038/s41467-022-28636-7 (PMC8854633; doi:10.1038/s41467-022-28636-7)
Supplement: Supplementary file 1 — Supplementary Information [file 41467_2022_28636_MOESM1_ESM.pdf]

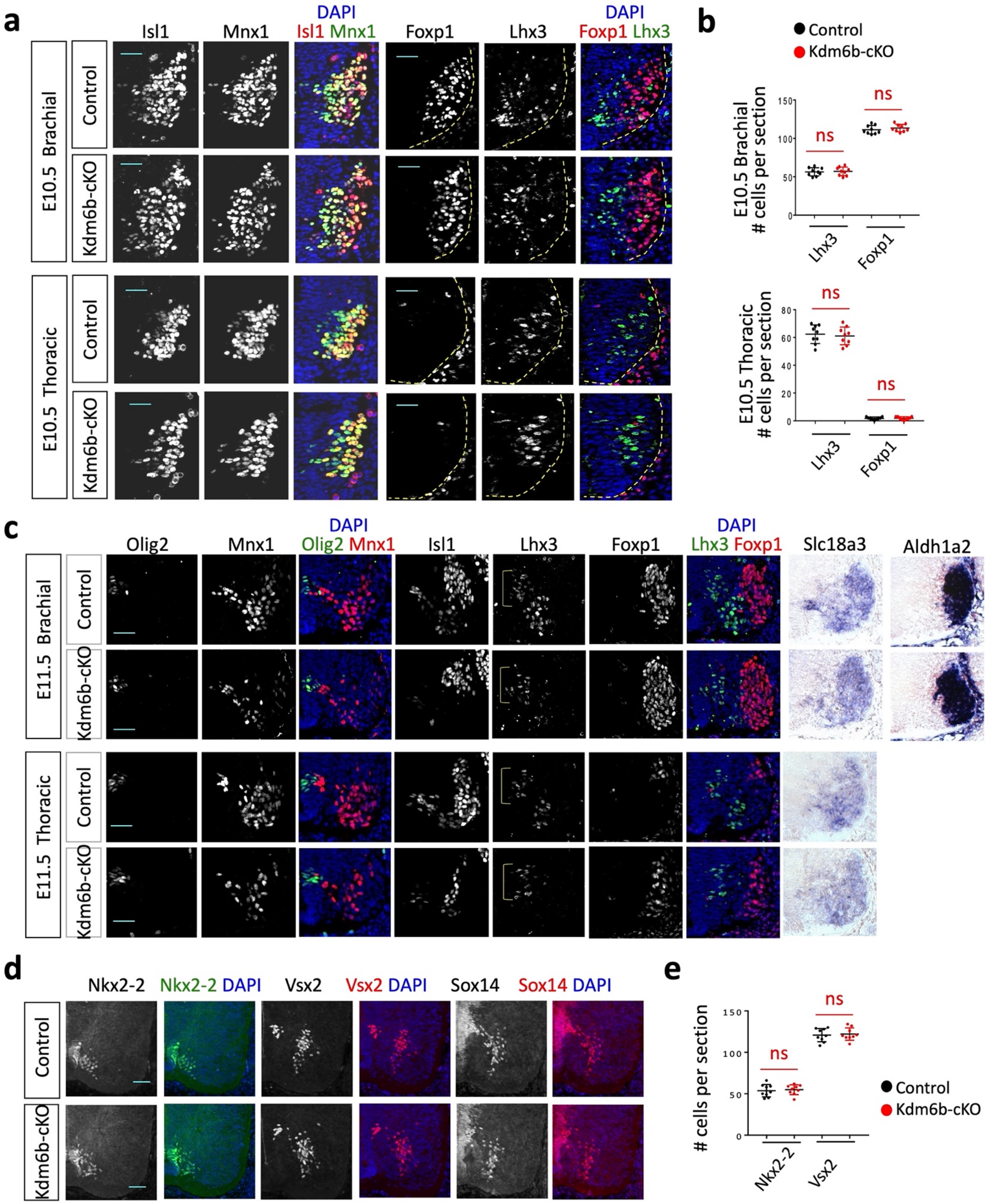

Supplementary Figure 1. Kdm6b was dispensable for early MN fate specification, but it was required for MN columnar diversification. (**a**) Immunohistochemical analyses with Isl1, Mnx1, Lhx3, and Foxp1 antibodies in E10.5 spinal cords at brachial and thoracic levels. $Isl1^{+}Mnx1^{+}$ MNs were comparable between control and *Kdm6b*-cKO at E10.5. Scale bars, 50 µm. (**b**) Quantification of the number of MNs expressing Lhx3 or Foxp1 per 12 µm thick section of the spinal cord at E10.5.

Neither Lhx3$^+$ nor Foxp1$^+$ MNs significantly changed in E10.5 *Kdm6b*-cKO spinal cords. The error bars represent the standard deviation of the mean. ns, non-significant in the two-tailed Student's t-test. n = 3-6 mice per genotype and 9 slices per genotype. (**c**) Immunohistochemical analyses with Olig2, Mnx1, Isl1, Lhx3, and Foxp1 antibodies and in situ hybridization analyses for Aldh1a2 and Slc18a3 in E11.5 spinal cords at brachial and thoracic levels. In E11.5 *Kdm6b*-cKO mice, the MNs expressing Mnx1, Isl1, or Lhx3 decreased, whereas the MNs expressing Foxp1 or Aldh1a1 increased. The brackets indicate Lhx3$^+$ V2 interneurons. Scale bars, 50 µm. (**d,e**) Immunohistochemical analyses with antibodies against p3 progenitor and V3 interneuron marker Nkx2-2 and V2a interneuron markers Vsx2 and Sox14 in E12.5 spinal cords. Scale bars, 50 µm. (**e**) Quantification of the number of Nkx2-2$^+$ (p3 progenitors and V3 interneurons) or Vsx2$^+$ (V2a interneurons) cells per 12 µm thick section. Nkx2-2$^+$ and Vsx2$^+$ cells were comparable between control and *Kdm6b*-cKO mice. The error bars represent the standard deviation of the mean. ns, non-significant in the two-tailed Student's t-test. n = 3-6 mice per genotype and 9 slices per genotype. (**a-e**) 3-6 embryos for each genotype and stage were analyzed and the representative images were shown. Source data for quantification are provided as a Source Data file.

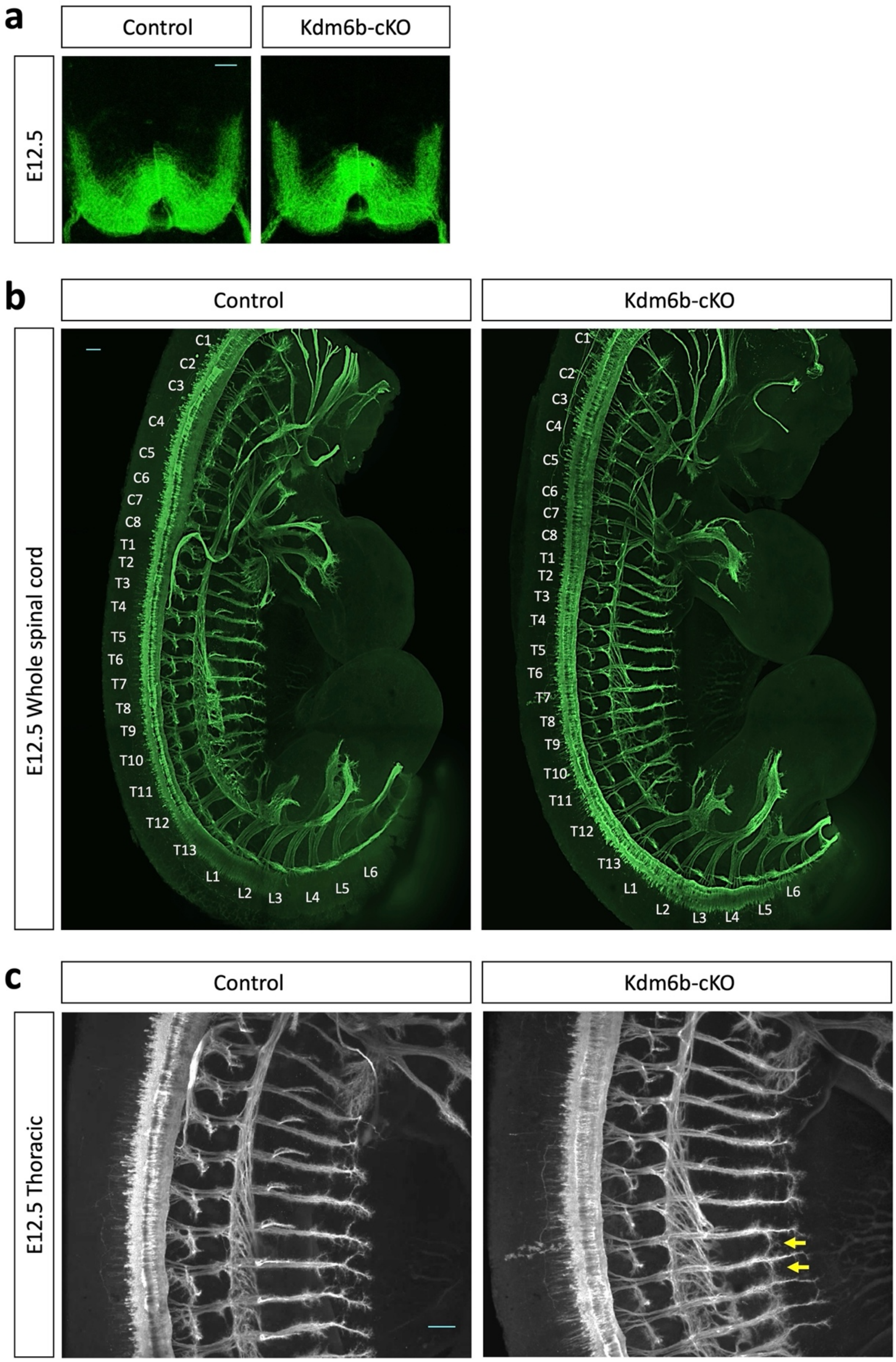

Supplementary Figure 2. Analysis of GFP$^+$ motor axonal projection pattern. (**a**) Transverse sections of E12.5 control (*Kdm6b$^{f/+}$;Olig2-Cre;mTmG*) and *Kdm6b*-cKO (*Kdm6b$^{f/f}$;Olig2-Cre;mTmG*) mice. mGFP is expressed specifically in *Olig2-Cre* lineage cells, including MNs, within the spinal cord. In the periphery, only motor axons leaving the spinal cord expressed mGFP. Scale bars, 100 µm. (**b,c**) The lateral view of E12.5 embryos to analyze GFP$^+$ motor axonal trajectory in the periphery. Scale bars, 100 µm. (**c**) The enlarged view of E12.5 embryos to analyze GFP$^+$ motor axonal trajectory at thoracic levels. In *Kdm6b-cKO* mice, HMC-derived GFP$^+$ motor axons begun to form abnormal bridge-like connections between intercostal nerves bundles (yellow arrows). Scale bars, 100 µm. (**a-c**) 3-5 embryos per genotype in each condition were analyzed and the representative images were shown.

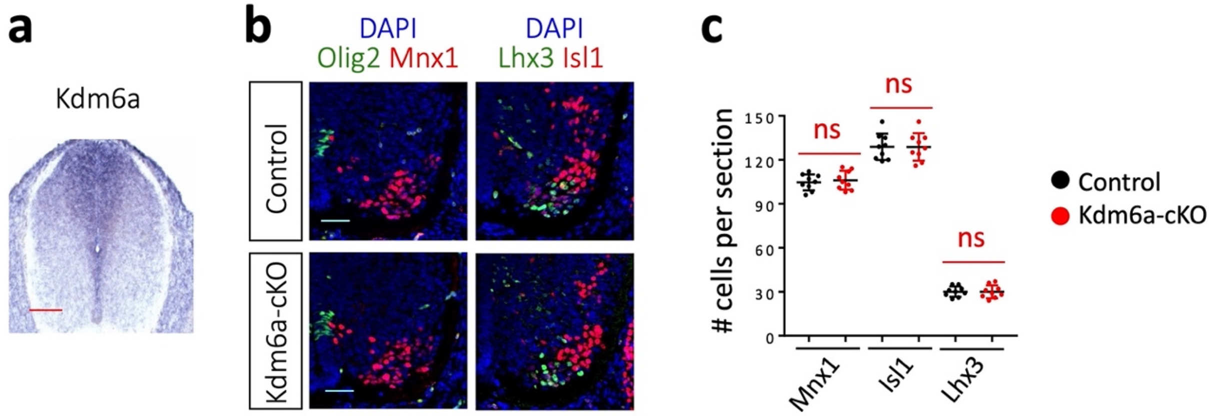

Supplementary Figure 3. Kdm6a was not needed for MN differentiation and MN columnar diversification. (**a**) The expression pattern of Kdm6a in E12.5 spinal cord, as detected by in situ hybridization analyses. Scale bars, 100 µm. (**b**) Immunohistochemical analyses with Olig2, Mnx1, Isl1, and Lhx3 antibodies in E12.5 control and *Kdm6a*-cKO embryo spinal cords at thoracic levels. Scale bars, 50 µm. (**c**) Quantification of the number of MNs expressing Mnx1, Isl1 or Lhx3 per 12 µm thick section of the thoracic level spinal cord at E12.5. The error bars represent the standard deviation of the mean. ns, non-significant in the two-tailed Student's t-test. n = 3-6 mice per genotype and 9 slices per genotype.

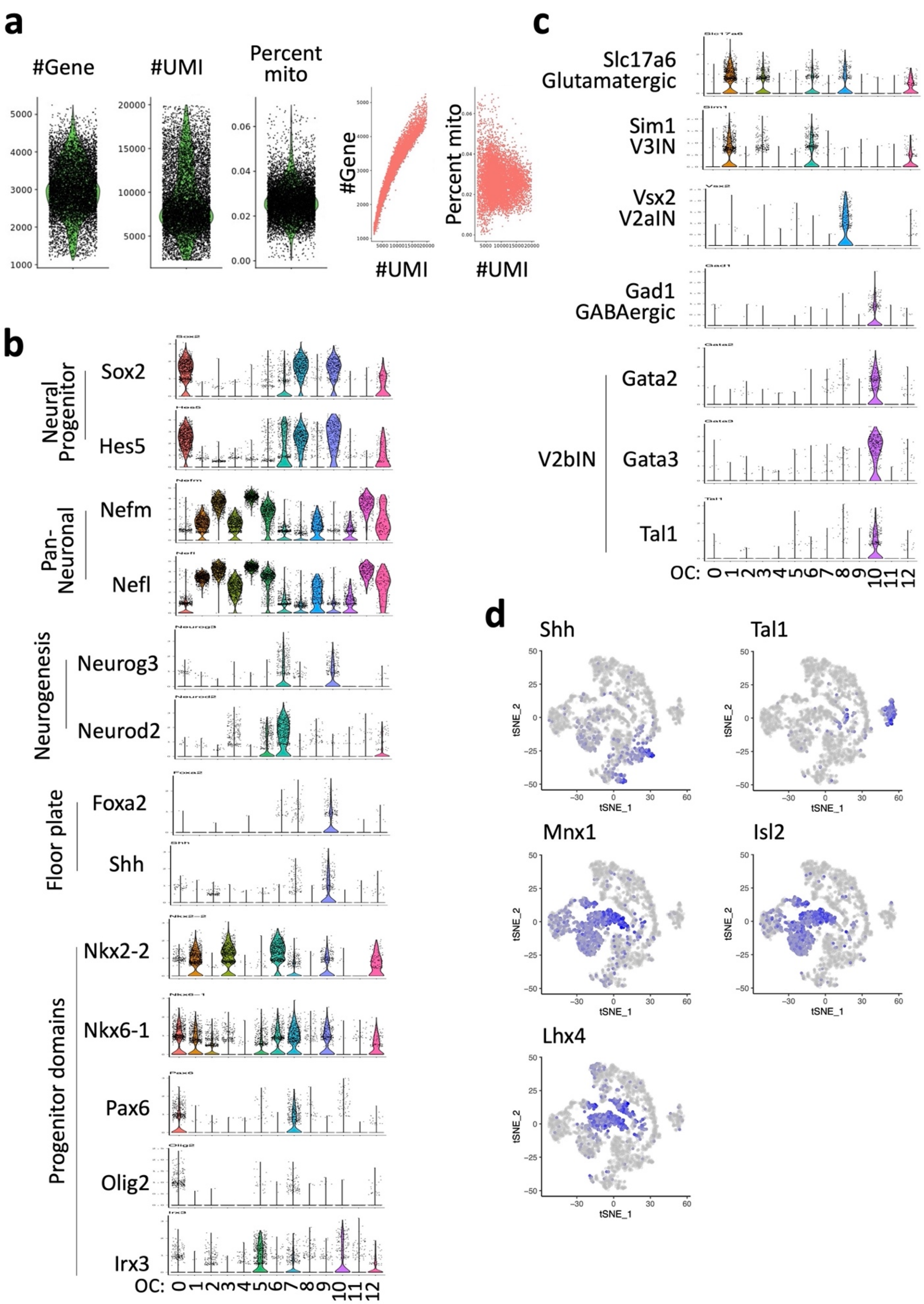

Supplementary Figure 4. Single-cell transcriptome analyses of *Olig2-Cre* lineage cells in E12.5 spinal cord. (**a**) Distribution of the number of genes, total number of Unique Molecular Identifier (UMI), and percentages of mitochondrial genes (Percent mito) within a cell for GFP^{+} control cell population that passed quality control. (**b,c**) Violin plots for selected marker genes in OC0-OC12. (**d**) tSNE plots to show the expression of selected marker genes. The expression levels of the genes were indicated by blue color intensi

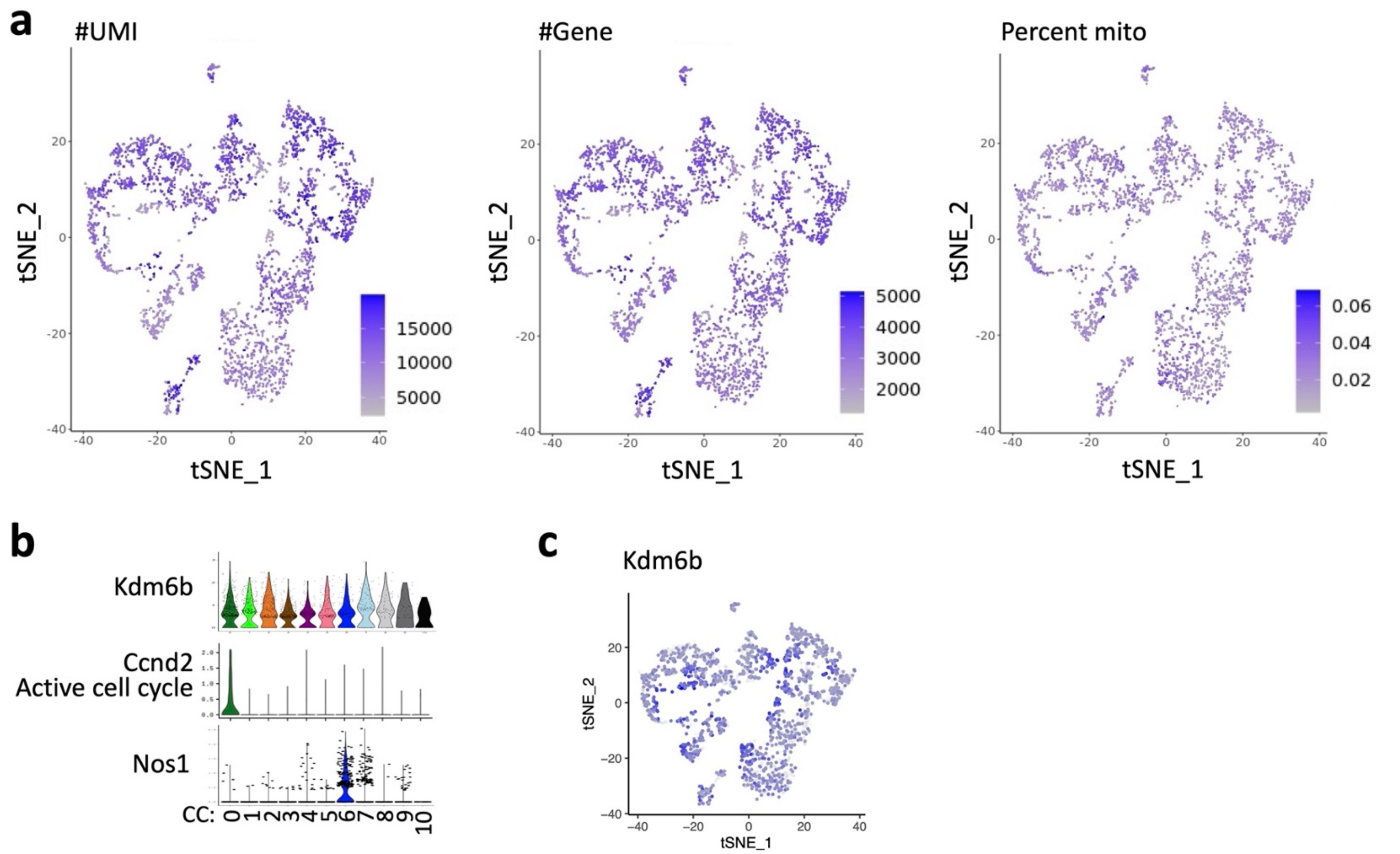

Supplementary Figure 5. Single-cell transcriptome analyses of Slc18a3⁺ MN lineage cells. (**a**) Visualization of quality control for Slc18a3⁺ control cell population to check for the presence of biases across the low-dimensional feature space according to the total number of UMI, number of genes, and percentages of mitochondrial genes within a cell. (**b**) Violin plots for selected marker genes in CC0-CC10. (**c**) tSNE plot to show Kdm6b expression. Kdm6b is broadly expressed in Slc18a3⁺ cells, as indicated by blue color intensity.

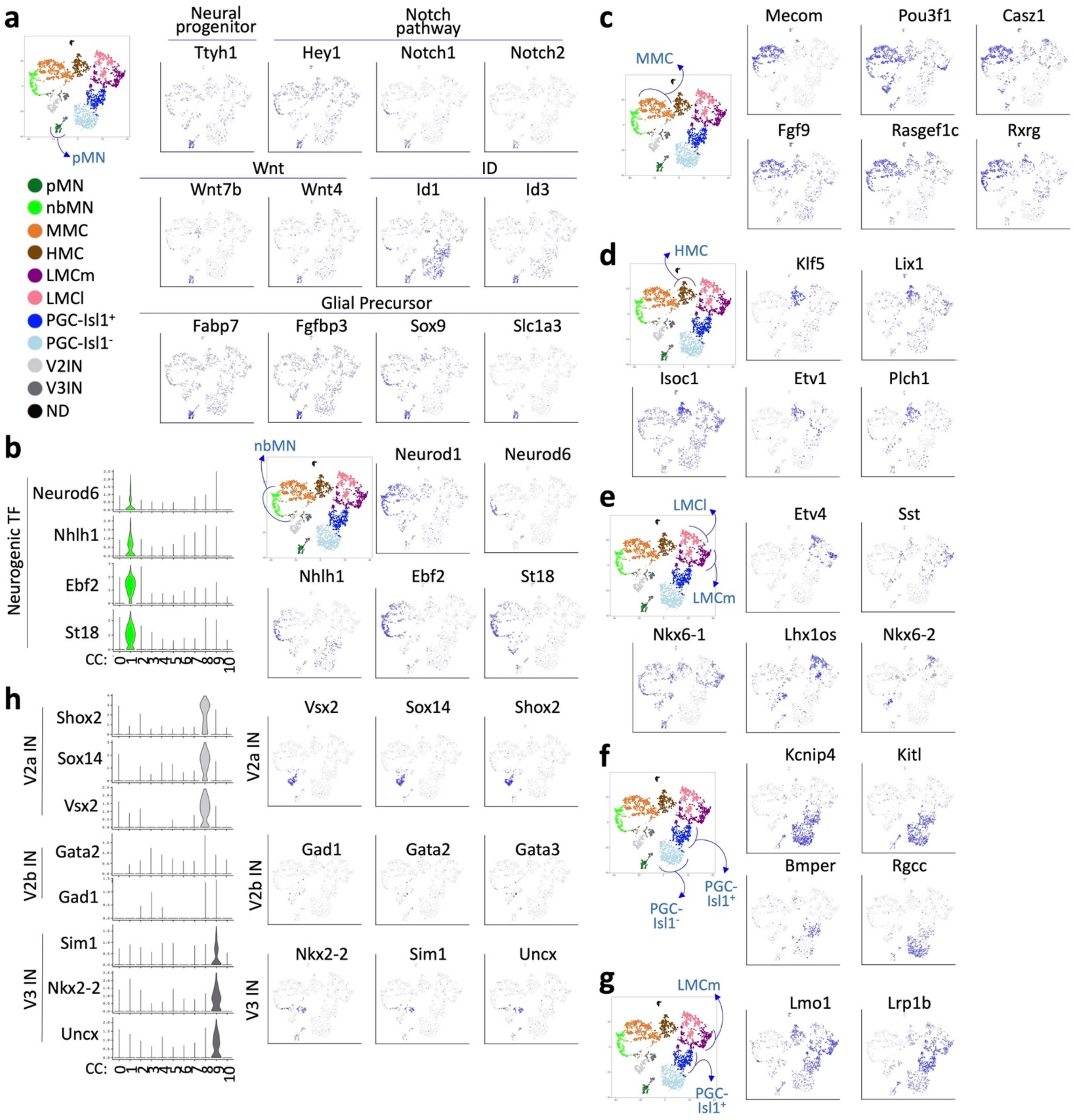

Supplementary Figure 6. Violin and tSNE plots to show the expression level and pattern of selected marker genes in control clusters CC0-CC10. Each dot represents a single cell. The expression levels of the genes in each dot were indicated by blue color intensity. The genes were selected from the marker list for pMN (**a**), nbMN (**b**), MMC (**c**), HMC (**d**), LMCl (**e**), PGC (**f**), LMCm and PGC-Isl1^{+} (**g**), and V2IN and V3IN (**h**) clusters.

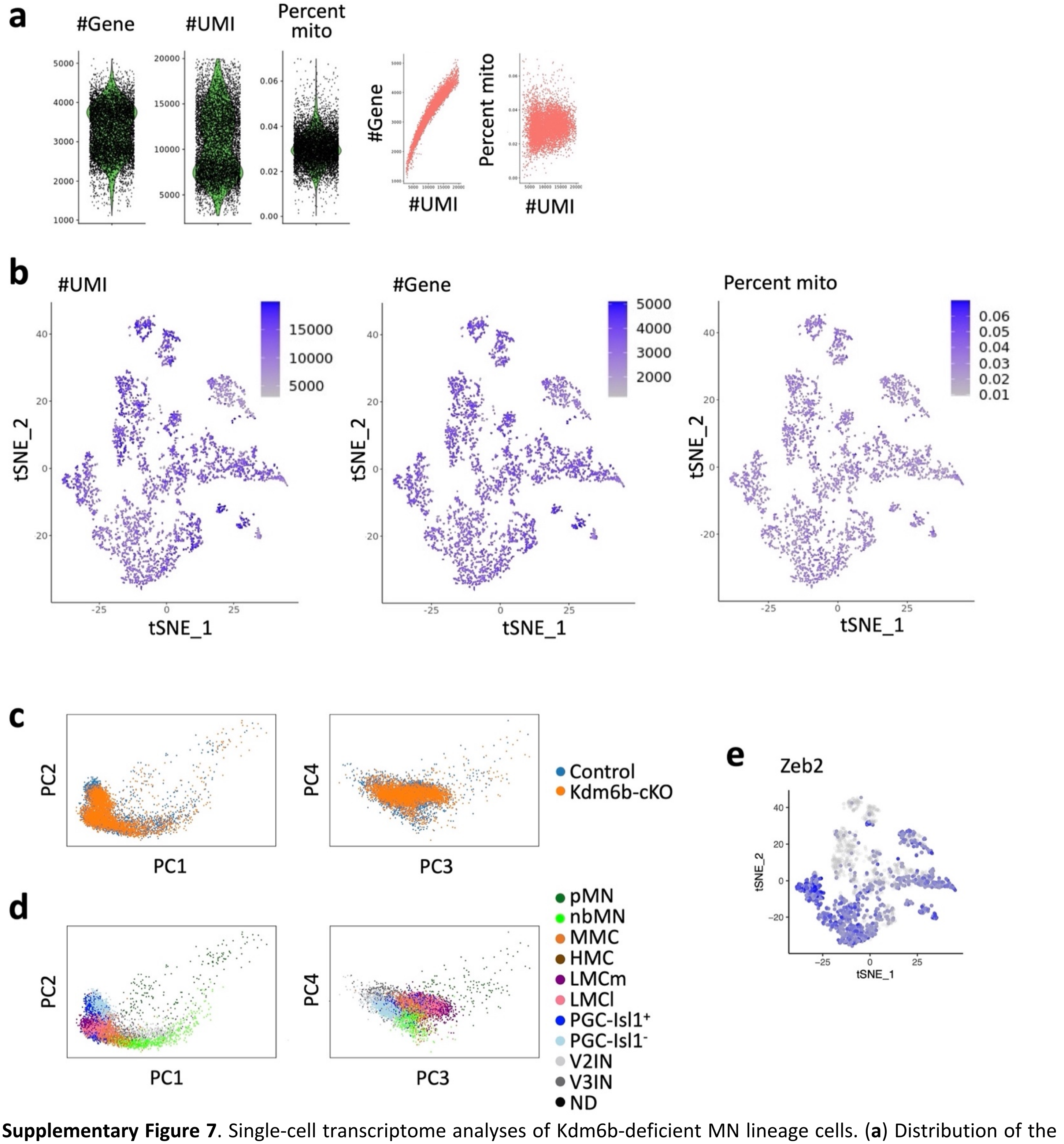

Supplementary Figure 7. Single-cell transcriptome analyses of Kdm6b-deficient MN lineage cells. (**a**) Distribution of the number of genes, total number of UMI, and percentages of mitochondrial genes within a cell for GFP^{+} *Kdm6b*-cKO cell population that passed quality control. (**b**) Visualization of quality control for Slc18a3^{+} *Kdm6b*-cKO population to check for the presence of biases across the low-dimensional feature space according to the total number of UMI, number of genes, and percentages of mitochondrial genes within a cell. (**c,d**) Projection of Slc18a3^{+} population of cells for both control and *Kdm6b*-cKO into the same PCA subspace (left panel, PC1-2; right panel, PC3-4) before Seurat (CCA) integration to check for the presence of batch effects. Each cell is color-coded by its conditions (c) and by cell types (d) as defined in Fig. 7a. There are few batch effects between control and *Kdm6b*-cKO samples. (**e**) tSNE plots show the expression level of Zeb2, as indicated by blue color intensity.

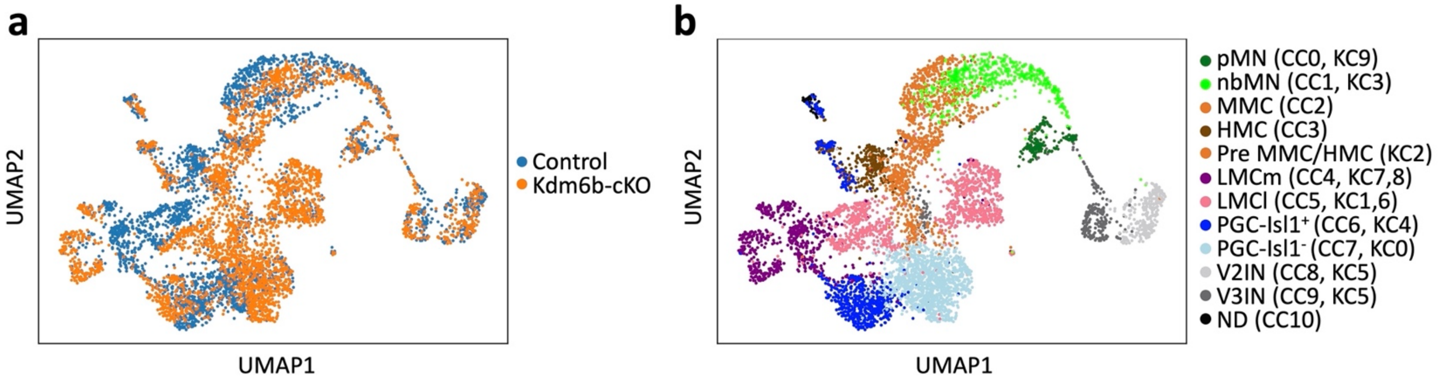

c

Cell identity	Control Cluster (CC) CC#	Cell #	KO Cluster (KC) KC#	Cell #
pMN	CC0	91	KC9	85
nbMN	CC1	224	KC3	353
MMC	CC2	533	-	-
HMC	CC3	293	-	-
Presumptive MMC/HMC	-	-	KC2	505
LMCm	CC4	397	KC8+KC7	251
LMCl	CC5	341	KC1+KC6	720
PGC-Isl1+	CC6	325	KC4	344
PGC-Isl1-	CC7	463	KC0	754
V2+V3IN	CC8+CC9	311	KC5	266
ND	CC10	42	-	-
Total		3020		3278

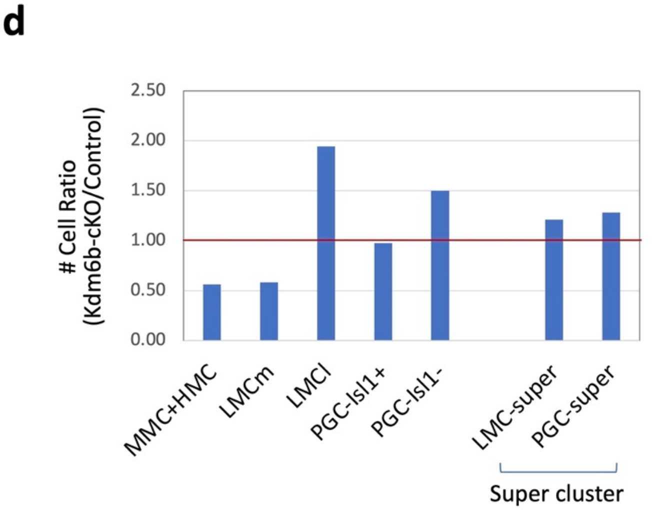

Supplementary Figure 8. (**a-c**) Projection of Slc18a3^{+} population of cells for both control and *Kdm6b*-cKO, without Seurat canonical correlation analysis (CCA) integration, into the same UMAP feature space. Control and *Kdm6b*-cKO cells in pMN, nbMN, V2IN, and V3IN clusters are relatively well intermingled, whereas MN subtype cells tend to show more segregated patterns between control and *Kdm6b*-cKO cells. Each cell is color-coded by its genotype (**a**) and cell types as defined in Fig. 4a (CC, control cluster) and 7a (KC, KO cluster). As there are no clear MMC and HMC clusters in the *Kdm6b*-cKO sample, the presumptive MMC/HMC cluster (pre MMC/HMC) was plotted using the MMC color code for the *Kdm6b*-cKO sample. (**c**) The merged table for Figure 4b and 6b. (**d**) The ratio of *Kdm6b*-cKO cells over control cells in each cell type cluster or "super-cluster" marked as LMC-super (LMCl and LMCm) or PGC-super (PGC-Isl1^{+} and PGC-Isl1^{-}).

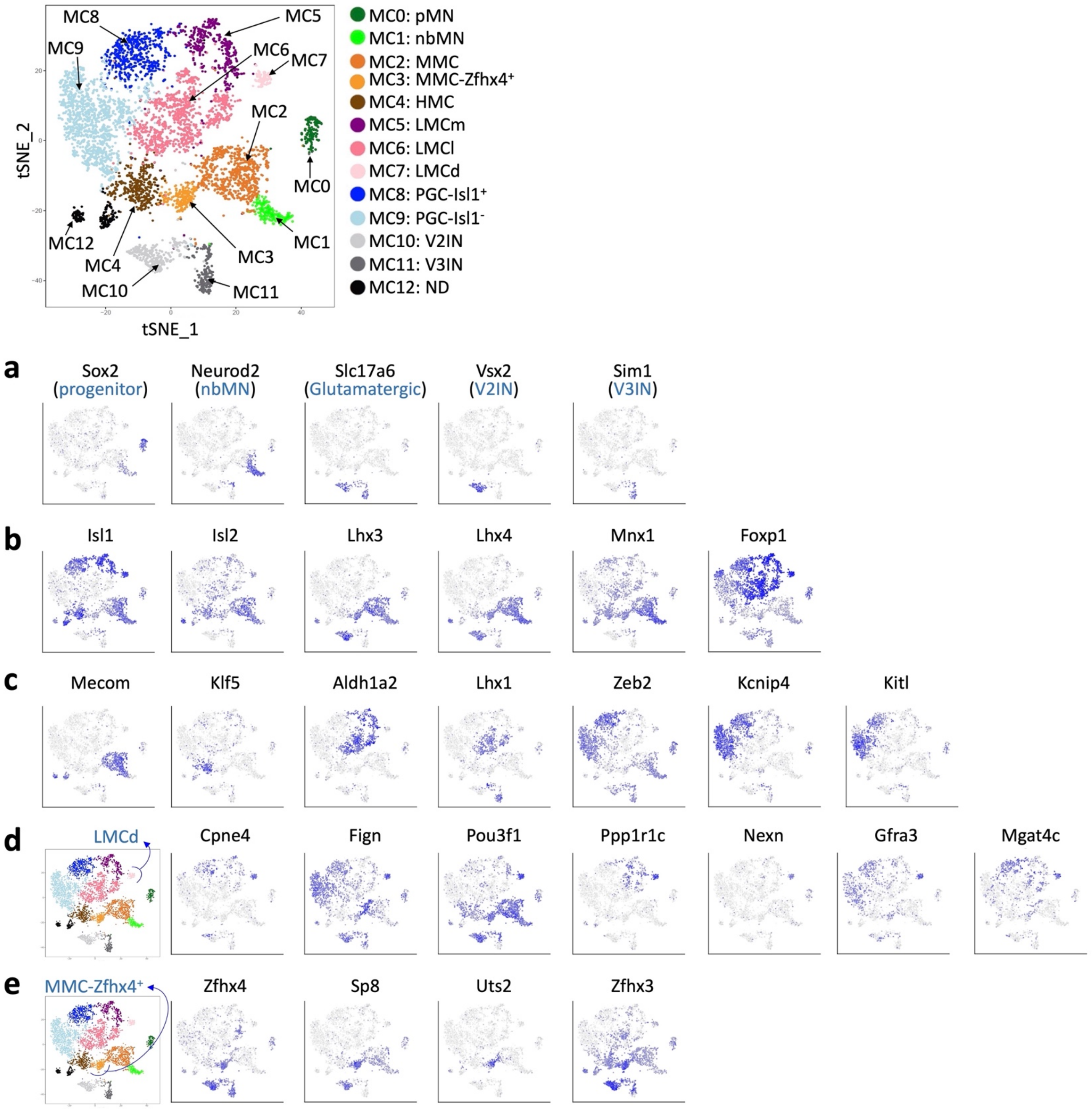

Supplementary Figure 9. tSNE plots show the expression level and pattern of selected marker genes in merged clusters MC0-MC12. Each dot represents a single cell. The expression levels of the genes in each dot were indicated by blue color intensity. The genes were selected for the genes enriched in immature cell type clusters (**a**), MN fate-specifying TFs (**b**), MN subtype marker genes (**c**), LMCd marker genes (**d**), and MMC-Zfhx4^{+} marker genes (**e**).

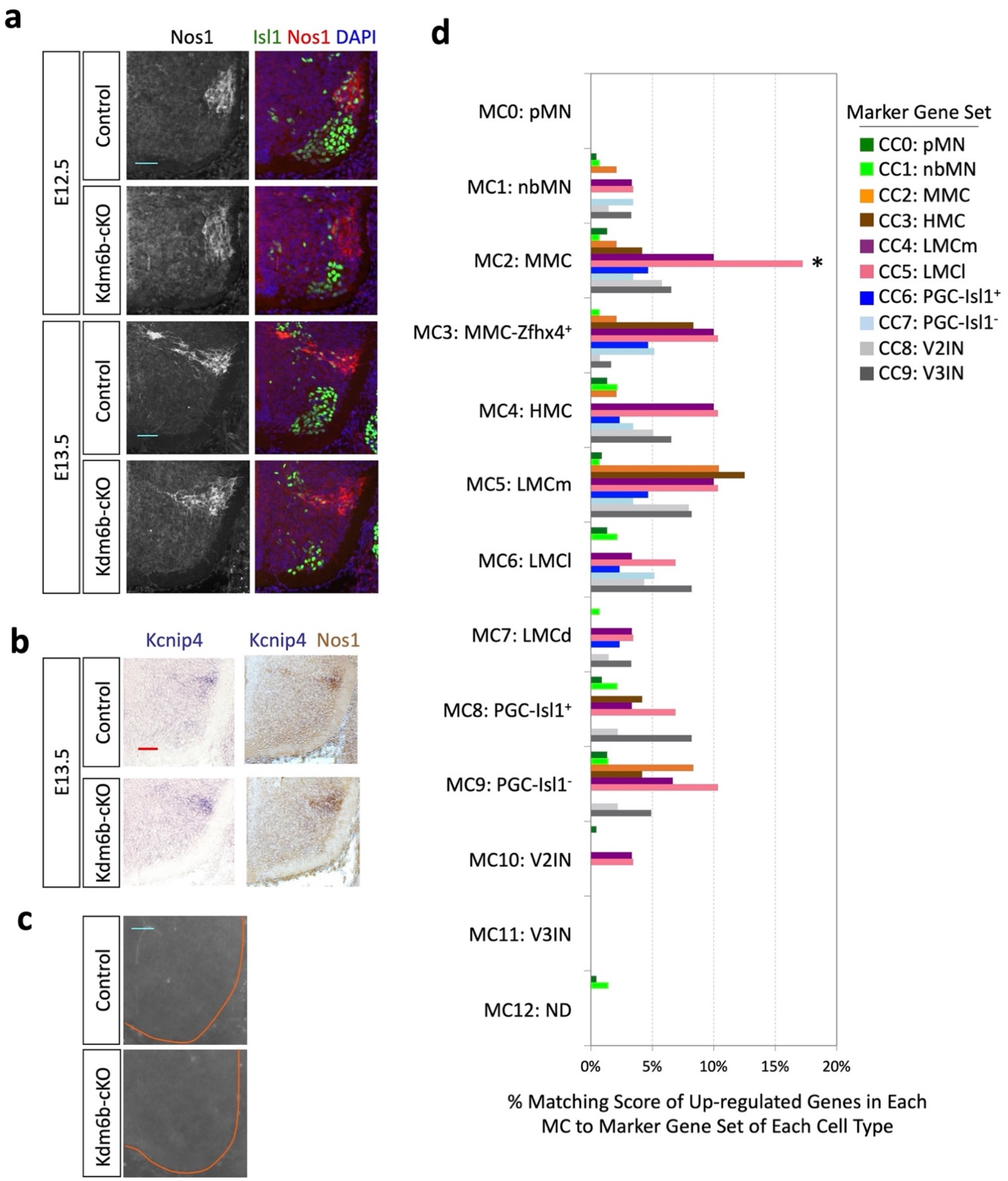

Supplementary Figure 10. Kdm6b regulates MN columnar diversification. (**a,b**) Immunohistochemical analyses with Isl1 and Nos1 antibodies (a) and in situ hybridization analyses for Kcnip4 and Nos1 (b) in thoracic spinal cords of *Kdm6b*-cKO and control mice. Kcnip4^{+}Nos1^{+} PGC MN area was expanded, but Isl1/Nos1 double-positive cells reduced in *Kdm6b*-cKO relative to control mice. Scale bars, 50 µm. (**c**) TUNEL assays revealed no significant change in apoptosis in E12.5 *Kdm6b*-cKO MNs. Scale bars, 50 µm. (**a-c**) 3-6 embryos per genotype in each condition were analyzed and the representative images were shown. (**d**) Pairwise comparison of the genes significantly up-regulated in each MC to the marker gene sets in the control datasets CC0-CC9 in Fig. 5a. Matching scores show the percentage of each MC's up-regulated genes among the marker gene sets in CC0-CC9. The up-regulated genes in Kdm6b-deficient MMC and the LMCl marker set showed the highest matching score of 17% (marked by an asterisk).

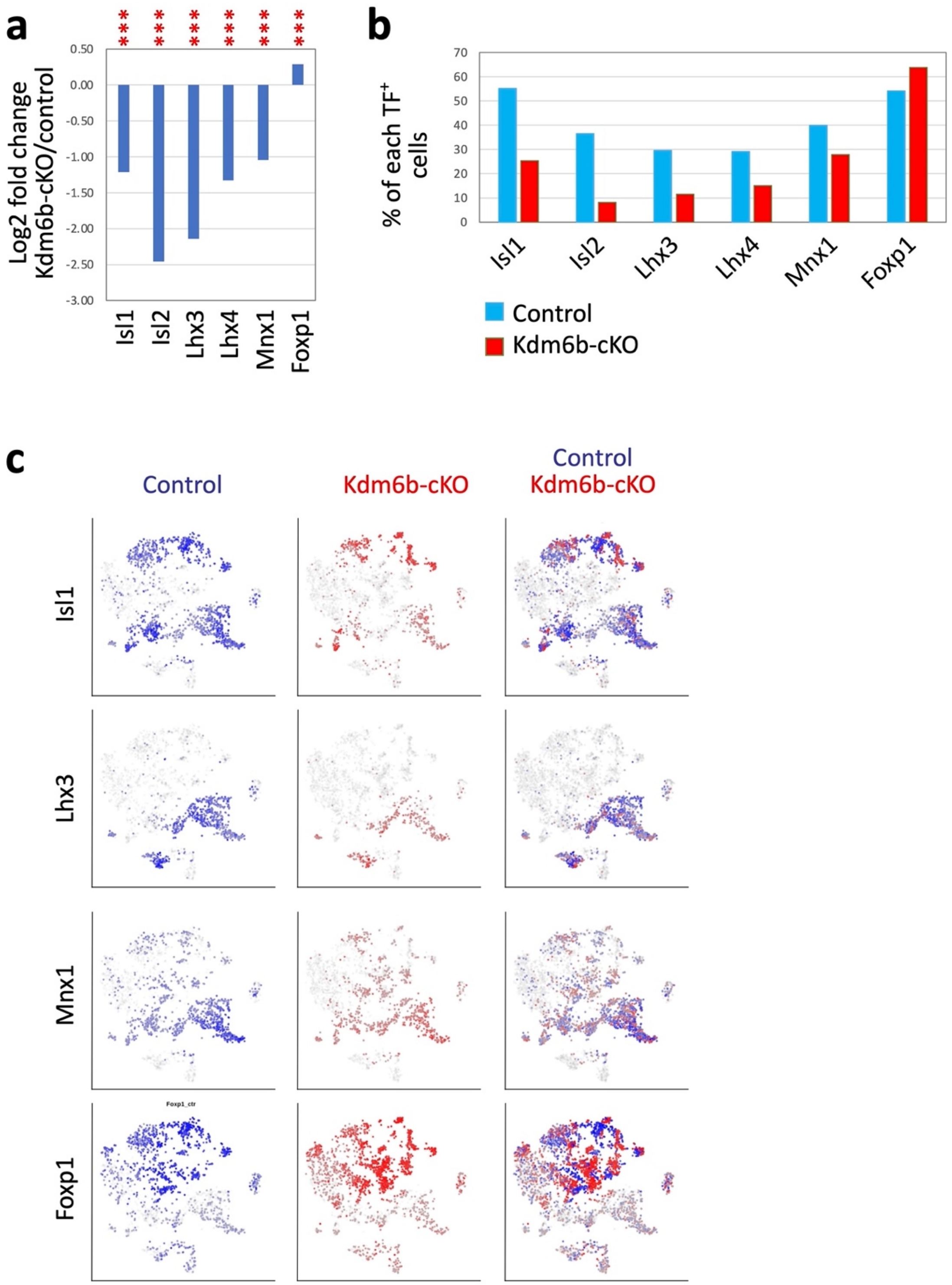

Supplementary Figure 11. Isl1/2, Lhx3/4, and Mnx1 genes show a remarkable down-regulation, whereas Foxp1 exhibits a significant up-regulation in Kdm6b-deficient MN lineage. (**a**) Relative gene expression levels of MN fate-specifying TFs in all Slc18a3^{+} cells in *Kdm6b*-cKO compared to the control sample. ***, *p.adj*<0.001. The *p*-value was calculated using the *WilcoxDETest* function in the Seurat package, in which a two-sided alternative hypothesis testing is performed. Multiple testing correction was carried out using the *p.adjust* function with *–fdr* option in the R package. The exact *p*-values are as follows. Isl1, $1.4x10^{-127}$; Isl2, $6.72x10^{-167}$; Lhx3, $2.21x10^{-79}$; Lhx4, $4.55x10^{-46}$; Mnx1, $1.11x10^{-31}$; Foxp1, $4.58x10^{-13}$. (**b**) The percentage of each TF-expressing cells among all analyzed Slc18a3^{+} cells in *Kdm6b*-cKO and control samples. (**c**) tSNE plots visualize the expression level and pattern of Isl1, Lhx3, Mnx1, and Foxp1 in merged datasets. Each dot represents a single cell. The expression levels of the genes in each dot were indicated by blue (in control cells) or red (in *Kdm6b*-cKO) color intensity. The first and second columns show the location of each marker-expressing cells from the control (blue) or *Kdm6b*-cKO (red) sample in separate tSNE plots for each genotype. The third column shows the location of each marker-expressing cells from both control (blue) and *Kdm6b*-cKO (red) samples in the same tSNE plot.
