# Supplementary material for: The histone demethylase Kdm6b regulates subtype diversification of mouse spinal motor neurons during development
Source: Nat Commun. 2022 Feb 17;13:958. doi: 10.1038/s41467-022-28636-7 (PMC8854633; doi:10.1038/s41467-022-28636-7)
Supplement: Supplementary file 3 — Description of Additional Supplementary Files [file 41467_2022_28636_MOESM3_ESM.docx]

**SUPPLEMENTARY DATA LEGENDS**

**Supplementary Data 1**. Marker gene lists for 11 control clusters CC0-CC10. The marker genes were specifically enriched in each cluster relative to the remaining clusters (FDR<0.01, log2fold change>0.4).

**Supplementary Data 2**. Directly comparison of the transcriptomes between the two LMC clusters, LMCm and LMCl, or between the two PGC clusters PGC-Isl1^+^ and PGC-Isl1^-^. Differentially expressed genes between the two clusters were selected with the cut-off of FDR<0.01 and the mean expression >0.04 to exclude the noise effect.

**Supplementary Data 3**. The list of genes that were significantly enriched in each of MC0-MC12 clusters. The listed genes were specifically enriched in each cluster relative to the remaining clusters (FDR<0.01, log2fold change>0.4).

**Supplementary Data 4**. The list of differentially expressed genes (DEG) between *Kdm6b*-cKO and control cells in each of MC0-MC12 clusters. The cut-off was set at FDR<0.01, absolute log2 fold change>0.8, and the mean expression >0.1 to exclude noise effect.
