# Supplementary material for: The histone demethylase Kdm6b regulates subtype diversification of mouse spinal motor neurons during development
Source: Nat Commun. 2022 Feb 17;13:958. doi: 10.1038/s41467-022-28636-7 (PMC8854633; doi:10.1038/s41467-022-28636-7)
Supplement: Supplementary file 8 — Reporting Summary [file 41467_2022_28636_MOESM8_ESM.pdf]

nature research

Corresponding author(s): Soo-Kyung Lee

Last updated by author(s): 2022/01/01

Reporting Summary

Nature Research wishes to improve the reproducibility of the work that we publish. This form provides structure for consistency and transparency in reporting. For further information on Nature Research policies, see our Editorial Policies and the Editorial Policy Checklist.

Statistics

For all statistical analyses, confirm that the following items are present in the figure legend, table legend, main text, or Methods section.

n/a	Confirmed	
☐	☒	The exact sample size (*n*) for each experimental group/condition, given as a discrete number and unit of measurement
☐	☒	A statement on whether measurements were taken from distinct samples or whether the same sample was measured repeatedly
☐	☒	The statistical test(s) used AND whether they are one- or two-sided<br>*Only common tests should be described solely by name; describe more complex techniques in the Methods section.*
☒	☐	A description of all covariates tested
☒	☐	A description of any assumptions or corrections, such as tests of normality and adjustment for multiple comparisons
☐	☒	A full description of the statistical parameters including central tendency (e.g. means) or other basic estimates (e.g. regression coefficient) AND variation (e.g. standard deviation) or associated estimates of uncertainty (e.g. confidence intervals)
☐	☒	For null hypothesis testing, the test statistic (e.g. *F*, *t*, *r*) with confidence intervals, effect sizes, degrees of freedom and *P* value noted<br>*Give P values as exact values whenever suitable.*
☒	☐	For Bayesian analysis, information on the choice of priors and Markov chain Monte Carlo settings
☒	☐	For hierarchical and complex designs, identification of the appropriate level for tests and full reporting of outcomes
☒	☐	Estimates of effect sizes (e.g. Cohen's *d*, Pearson's *r*), indicating how they were calculated

Our web collection on statistics for biologists contains articles on many of the points above.

Software and code

Policy information about availability of computer code

Data collection	We provided all the information in "methods".
Data analysis	10X Genomics Cellranger v.2.1.1 was used to process raw sequencing data, in which STAR v2.4.0 was used for the sequencing alignment. Seurat package v3.1.4 was used for scRNA-seq analysis in conjunction with R v4.0.2 and ggplot2 v3.3.5. For UMAP projection analysis, we used python packages pandas v1.1.2, Scanpy v1.6.0, and anndata v0.7.6.<br>CODE AVAILABILITY: Exemplary scripts to process and analyze data are available at https://github.com/epigenomekdm6b/kdm6b.

For manuscripts utilizing custom algorithms or software that are central to the research but not yet described in published literature, software must be made available to editors and reviewers. We strongly encourage code deposition in a community repository (e.g. GitHub). See the Nature Research guidelines for submitting code & software for further information.

Data

Policy information about availability of data

All manuscripts must include a data availability statement. This statement should provide the following information, where applicable:

- Accession codes, unique identifiers, or web links for publicly available datasets
- A list of figures that have associated raw data
- A description of any restrictions on data availability

The datasets generated during and/or analyzed during this study have been deposited in the Gene Expression Omnibus (GEO) under accession number “GSE156609 [https://www.ncbi.nlm.nih.gov/geo/query/acc.cgi?acc=GSE156609]”. The plasmids and antibodies are available upon request. A reporting summary for this article is available as a Supplementary Information file. Source data are provided with this paper.

Field-specific reporting

Please select the one below that is the best fit for your research. If you are not sure, read the appropriate sections before making your selection.

- [x] Life sciences
- [ ] Behavioural & social sciences
- [ ] Ecological, evolutionary & environmental sciences

For a reference copy of the document with all sections, see nature.com/documents/nr-reporting-summary-flat.pdf

Life sciences study design

All studies must disclose on these points even when the disclosure is negative.

Sample size	Unpaired two-tailed Student's t-test was used for statistical quantification. According to the standard and previous experience in the field, we picked up at least 3 mouse embryos for each genotype by testing genotypes from mice collected at indicated stages . Then at least 3 cryosections for each animal were chosen for immunostaining or in situ hybridization analyses, followed by quantification as needed. We used 9 datasets from each genotype for quantification, which is rational for statistical analysis. Increasing sample size didn't change the conclusion.
Data exclusions	For scRNAseq analysis, sequencing data were filtered according to standard Seurat analysis criteria as follows. Raw sequencing cell barcodes were filtered to distinguish valid cell barcodes from empty cell barcodes using an algorithm in Cellranger count v2.1.1, which is an analysis pipeline for Chromium single cell 3’ RNA-seq result with an expected recovered cell parameter; expect-cells set to 9,000 and 7,800 for control and Kdm6b-cKO, respectively. The gene-barcode count matrix generated was read using the Read10X function in Seurat v3.1.4. Initially, we kept all genes expressed in >3 cells and all cells with at least 200 detected genes. To exclude cells in poor quality and possible doublets, possible outliers of cells were further removed by visual inspection of the distribution of a total number of mapped genes. Cells with a high level of mitochondrial genes (>7%) were also removed, which were treated as mitochondrial cytoplasmic RNAs, which were released and sequenced due to cell lysis. Cells with a high level of hemoglobin gene expression (Hba-a1>10) were also removed, as they are likely to represent rare blood cell contaminants.<br>For other analyses, no data were excluded.
Replication	All mouse analyses, including immunostaining and in situ hybridization assays and associated quantifications, were performed on at least three independent animals and at least three sections for each animal, with data from individual replicates and the mean presented. We repeated the animal experiments every year for three years and all results were reproducible. For scRNAseq, co-immunoprecipitation, chromatin immunoprecipitation, and luciferase experiments, at least one more independent experimental set was performed for each test, and all produced consistent results, indicating good reproducibility.
Randomization	For the experiments involving cell culture work, independent experiments were randomized by choosing different days and transfecting the different cell batches for each set of experiments. Randomization is not relevant to our mouse analyses as there was no additional treatment step for the animals. Control and Kdm6b-cKO mice were identified by their genotypes and subsequently analyzed in parallel for each litter. We analyzed multiple litters for the presented data to avoid any data resulting from litter-to-litter variations.
Blinding	Blinding was strictly used during data collection and analysis.

Reporting for specific materials, systems and methods

We require information from authors about some types of materials, experimental systems and methods used in many studies. Here, indicate whether each material, system or method listed is relevant to your study. If you are not sure if a list item applies to your research, read the appropriate section before selecting a response.

Materials & experimental systems

n/a	Involved in the study
[ ]	[x] Antibodies
[ ]	[x] Eukaryotic cell lines
[x]	[ ] Palaeontology and archaeology
[ ]	[x] Animals and other organisms
[x]	[ ] Human research participants
[x]	[ ] Clinical data
[x]	[ ] Dual use research of concern

Methods

n/a	Involved in the study
[x]	[ ] ChIP-seq
[x]	[ ] Flow cytometry
[x]	[ ] MRI-based neuroimaging

Antibodies

Antibodies used	The primary antibodies include goat anti-Olig2 (R&D Systems AF2418, 1:500), rabbit anti-Olig2 (Millipore AB9610, 1:1000), guinea pig anti-Mnx1 (Homemade, 1:500), rabbit anti-Isl1 (Abcam ab109517, 1:250), guinea pig anti-Isl1 (Homemade, 1:2000), rabbit anti-Lhx3 (Abcam ab14555, 1:1000), guinea pig anti-Lhx3 (Homemade, 1:250), guinea pig anti-Vsx2 (Homemade, 1:1000)47, guinea pig anti-Sox14 (Homemade, 1:500)21, rabbit anti-Nkx2-2, (Abcam ab191077, 1:50), rabbit anti-Foxp1 (Abcam ab16645, 1:2000), rabbit anti-

	Nos1 (Immunostar 24287, 1:1000), rabbit anti-Kdm6b (Novus Biologicals NBP1-06640, 1:100), guinea pig anti-Klf576, rabbit anti-Mecom (Cell Signaling Technology #2593, 1:500), sheep anti-Zfhx3 (Novus Biologicals AF7384, 1:200), rabbit anti-Zfhx4 (Sigma HPA023837, 1:200) and rabbit anti-GFP (Abcam ab290, 1:500). The secondary antibodies from Jackson ImmunoResearch Laboratories include donkey anti-goat Alexa Fluor 488(Cat.705- 545-147, 1:500), donkey anti-guinea pig Alexa Fluor 488 (Cat.706-545-148, 1:500), donkey anti-rabbit Alexa Fluor 488(Cat.711-545-152, 1:500), donkey anti-goat Alexa Fluor 594(Cat.705-585-147, 1:500), donkey anti-guinea pig Alexa Fluor 594(Cat.706-585-148, 1:500), and donkey anti-rabbit Alexa Fluor 594 (Cat.711-585-152, 1:500).
Validation	The commercially available primary antibodies in this study have been chosen based on recommendations on the manufacturer's website, which includes citations, applications, and species reactivity.<br>The homemade antibodies have been reported previously (references 13, 14, 18, 19, 27, 47). They were validated by immunostaining on mouse and chick embryonic spinal cords and confirming that each of these antibodies displays the expected staining pattern in the developing spinal cord of wild-type animals. As the expression pattern of the transcription factors in the developing spinal cord was well established in the field for the last two decades, it is straightforward to test the validity of antibodies using this method.

Eukaryotic cell lines

Policy information about cell lines

Cell line source(s)	HEK293 was obtained from ATCC (Cat. CRL-1573).
Authentication	HEK293 cell line was not authenticated.
Mycoplasma contamination	The cell line was tested for negative for mycoplasma contamination.
Commonly misidentified lines (See ICLAC register)	No commonly misidentified cell lines were used.

Animals and other organisms

Policy information about studies involving animals; ARRIVE guidelines recommended for reporting animal research

Laboratory animals	The mice were in purified C57BL/6 or C57BL/6 mixed background. The mice were housed at 12 hour dark/12 hour light cycle, 25°C, and 50% humidity conditions. The species, number and age of animals in every experiment were provided in figure legends. As all our analyses were performed in embryonic stages and there are no sex-dependent differences in embryonic motor neuron development, sex was not described.
Wild animals	The study didn't involve wild animals.
Field-collected samples	No samples were from the field.
Ethics oversight	All housing and analyses were approved by the University at Buffalo Institutional Animal Care and Use Committee (IACUC) (protocol number, 201900078).

Note that full information on the approval of the study protocol must also be provided in the manuscript.
